# Development and evaluation of a virtual microscopy application for automated assessment of Ki-67 expression in breast cancer

**DOI:** 10.1186/1472-6890-11-3

**Published:** 2011-01-25

**Authors:** Juho Konsti, Mikael Lundin, Heikki Joensuu, Tiina Lehtimäki, Harri Sihto, Kaija Holli, Taina Turpeenniemi-Hujanen, Vesa Kataja, Liisa Sailas, Jorma Isola, Johan Lundin

**Affiliations:** 1 FIMM - Institute for Molecular Medicine Finland, University of Helsinki, Helsinki, Finland; 2 Department of Oncology, Helsinki University Central Hospital, Helsinki, Finland; 3 Molecular and Cancer Biology Research Program, Biomedicum Helsinki, University of Helsinki, Helsinki, Finland; 4 University of Tampere and Department of Oncology, Tampere University Hospital, Tampere, Finland; 5 Department of Oncology and Radiotherapy, Oulu University Central Hospital, Oulu, Finland; 6 Department of Oncology, Kuopio University Hospital, Kuopio, Finland; 7 Department of Oncology, Vaasa Central Hospital, Vaasa, Finland; 8 Institute of Medical Technology, University of Tampere, Tampere, Finland; 9 Division of Global Health, Karolinska Institutet, Stockholm, Sweden

## Abstract

**Background:**

The aim of the study was to develop a virtual microscopy enabled method for assessment of Ki-67 expression and to study the prognostic value of the automated analysis in a comprehensive series of patients with breast cancer.

**Methods:**

Using a previously reported virtual microscopy platform and an open source image processing tool, ImageJ, a method for assessment of immunohistochemically (IHC) stained area and intensity was created. A tissue microarray (TMA) series of breast cancer specimens from 1931 patients was immunostained for Ki-67, digitized with a whole slide scanner and uploaded to an image web server. The extent of Ki-67 staining in the tumour specimens was assessed both visually and with the image analysis algorithm. The prognostic value of the computer vision assessment of Ki-67 was evaluated by comparison of distant disease-free survival in patients with low, moderate or high expression of the protein.

**Results:**

1648 evaluable image files from 1334 patients were analysed in less than two hours. Visual and automated Ki-67 extent of staining assessments showed a percentage agreement of 87% and weighted kappa value of 0.57. The hazard ratio for distant recurrence for patients with a computer determined moderate Ki-67 extent of staining was 1.77 (95% CI 1.31-2.37) and for high extent 2.34 (95% CI 1.76-3.10), compared to patients with a low extent. In multivariate survival analyses, automated assessment of Ki-67 extent of staining was retained as a significant prognostic factor.

**Conclusions:**

Running high-throughput automated IHC algorithms on a virtual microscopy platform is feasible. Comparison of visual and automated assessments of Ki-67 expression shows moderate agreement. In multivariate survival analysis, the automated assessment of Ki-67 extent of staining is a significant and independent predictor of outcome in breast cancer.

## Background

With the emergence of virtual microscopy and whole slide scanning techniques, there is an increasing need for efficient tools to automate assessment of digitized biological samples. One possible solution is to integrate computer vision methods with a virtual microscopy platform and to run the image analysis software on the same server system as the virtual slides are stored.

A considerable number of published scientific studies have addressed computer vision for quantification of protein expression as determined by immunohistochemistry (IHC) [1-16]. Only one of the previous studies is based on an open source solution [17]. Very few studies have compared human visual interpretation and computer vision of IHC expression levels with regard to clinically important endpoints, such as disease outcome [2,15,16].

While tissue sample processing and IHC staining methods are increasingly automated, the evaluation of staining results is still predominantly performed by visual assessment. A human interpreter has excellent image comprehension and pattern recognition capabilities, but is prone to substantial variability in quantification tasks. Computer vision methods are capable of processing images consistently and generally perform well in repetitive processes. Virtual microscopy combined with computer vision techniques can aid the human observer by analysis of large tissue areas at a high magnification. The digital sample (i.e. the virtual slide) can be an entire section of a single cancerous tumour or an array of 100-200 tumour tissue samples assembled by the use of tissue microarray technology [18].

We decided to develop and study a computer vision method for IHC analysis that can be run on a virtual microscopy platform and to compare the method to visual interpretation of IHC staining. A highly studied biomarker, Ki-67, with known prognostic value in many cancer forms was chosen as the target [9,11,12,19-22]. Ki-67 is a protein associated with cell proliferation and is present in all other cell cycle phases except G0, the resting phase. Ki-67 is thoroughly studied in breast cancer and Ki-67 immunostaining shown to be evaluable with computer vision methods [9,11,12]. One previous study found that semi-automated analysis of Ki-67 staining with image analysis can be used for prognostic assessment of patients with breast cancer [10].

In this study, a tool for automated quantitative assessment of Ki-67 expression is presented. The tool is implemented within a previously described web-based virtual microscopy platform [23]. The IHC quantification method is evaluated by comparing the results with visual assessment of Ki-67 expression in a comprehensive series of breast cancer specimens. By linking the clinicopathological data with related tissue samples, the relationship between automated Ki-67 expression analysis and survival is assessed.

## Methods

### Patients

The FinProg series consist of 2842 breast cancer patients diagnosed during 1991 and 1992 within five geographical regions of Finland. The regions cover half of the population and the cases represent 53% of all breast cancers diagnosed in Finland during this period. Clinical data associated with subjects were extracted from the hospital case records, hospital registries, the Finnish Cancer Registry, and Statistics Finland. The data comprises more than 50 clinicopathological factors, including the histological type and grade of breast cancer, the number of metastatic and examined lymph nodes, primary tumour size, tumour ER and PR content evaluated by immunohistochemistry in the TMA samples, treatment details, and follow-up data. More than 50 pathologists performed histological typing and grading of cancer at the time of the diagnosis according to the World Health Organization guidelines. The median follow-up time of subjects included in the study was 9.5 years. Permission to use clinical data and formalin-fixed, paraffin-embedded tissues for research purposes was provided by the Ministry of Social Affairs and Health, Finland (permission 123/08/97). With reference to the large number of studied cases the authorities granted permission to use tissue samples without individual patient consent.

### Exclusion criteria

**Figure 1:**
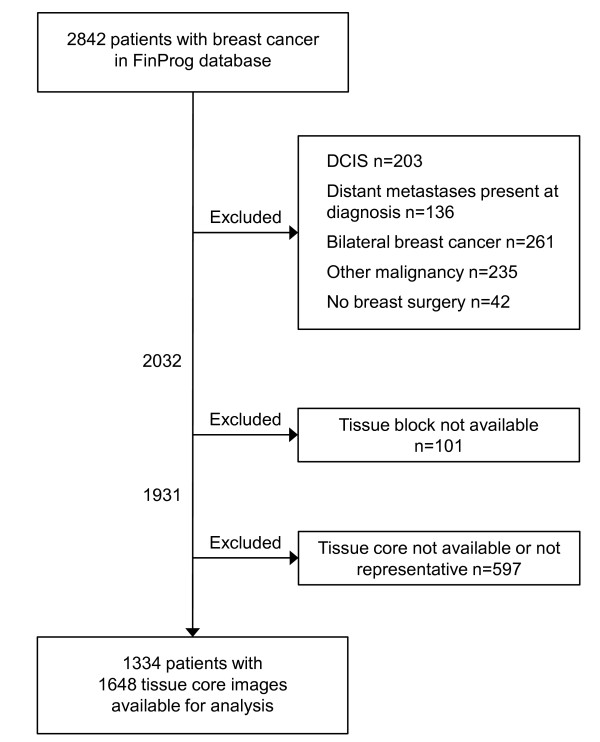
**Consort diagram**.

Subjects diagnosed with ductal or lobular carcinoma in situ were excluded from the study before statistical analyses as well as those who had distant metastases at the time of the diagnosis, bilateral breast cancer, or other malignancy than breast cancer in history, except basal cell carcinoma or cervical carcinoma in situ. Since the risk of dying from these last mentioned two malignant diseases after diagnosis and proper treatment is exceedingly small, the possible confounding effect on the survival estimates was considered insignificant and not to require exclusion of the subjects. A subject was also excluded if no breast surgery was carried out. A single subject may have been excluded for one or more reasons. A consort diagram is provided in figure 1.

### Preparation of tissue microarrays

Formalin-fixed, paraffin-embedded samples of the primary tumours (n = 1931) were collected and 1-4 tissue cores (core diameter 0.6 mm) from each patient assembled into tissue microarrays (TMAs, n = 23) as described elsewhere [24]. Sections of 5 μm were cut and processed for immunohistochemistry (IHC).

### Immunohistochemistry (IHC)

Immunostaining for Ki-67 was done using a mouse monoclonal antibody MM-1 (Novocastra Laboratories; 1:1,000 dilution) as previously described [25]. An evaluable Ki-67 staining was available in 1334 (69,1%) of the 1931 eligible cases with tumour tissue available for analysis. The number of samples lost due to tissue processing or with non-representative tissue spots was 597. The extent of Ki-67 staining was assessed visually in 1292 of the cases by one of the researchers (HS) under supervision of a single pathologist (JI) as part of a previous study [25], counting the number of positive tumour cells and classified into negative, moderate and high expression with cut-off values at 0% and 20%. Immunostainings for ER, PR, HER1, HER2, TP53, cyclooxygenase-2, KIT, GATA-3 and CK 5/6 were also assessed as previously reported [25]. Molecular subtypes were defined as luminal A (ER+ and/or PR+, HER2-), luminal B (ER+ and/or PR+, HER2+), basal-like (ER-, PR-, HER2-, cytokeratin 5+, and/or HER1+), HER2+/ER- (ER-, PR-, and HER2+), and five-marker negative (negative for all markers) [25].

### Sample digitization

Twenty-three TMA slides, with 2749 tissue spots including duplicates, triplicates and quadruplicates, were digitized with an automated whole-slide scanner (Mirax Scan, Zeiss, Göttingen, Germany), using a 20× objective and a DFW-X710 camera (Sony, Tokyo, Japan). The pixel resolution was 0.26 micrometers per pixel. The virtual slides were compressed to a wavelet file format (Enhanced Compressed Wavelet, ECW, ER Mapper, Erdas Inc, Atlanta, Georgia) with a conservative compression ratio of 1:5.

### Virtual microscopy platform

The computer vision algorithm was integrated with a previously described virtual microscopy platform [23,26], which allows image analysis scripts to be run in a batch mode on the server hosting the virtual slides. The compressed virtual slides of the TMAs stained for Ki-67 analysis were uploaded to the web server equipped with 2 quad-core Intel Xeon processors and 16 GB of RAM. TMA spot locations on the virtual slides were defined by an annotation system in the virtual microscopy user interface and linked to the corresponding clinical data. Images of the TMA spots, 1634×1634 pixels in size, were extracted from the virtual slides as separate image files, exported into the computer vision algorithm, and stored on the server for later manual inspection and documentation purposes.

### Computer vision algorithm

**Figure 2:**
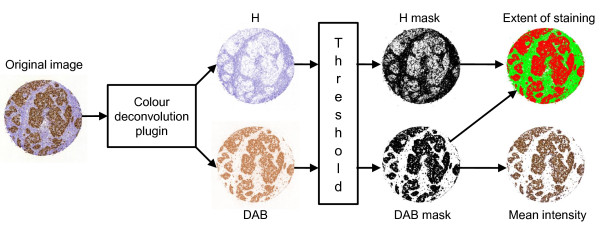
**The IhcJ algorithm for automatic quantitative assessment of Ki-67 proliferation index**.

The computer vision algorithm (IhcJ) is depicted in figure 2. It utilizes the macro language of an image processing and analysis software, ImageJ, which is open source and available free of charge for multiple operating systems at http://rsb.info.nih.gov/ij/. The IhcJ algorithm first divides the acquired image of the IHC stained specimen in RGB colour space into separate colour channels by a colour deconvolution method [27]. The ImageJ plugin for colour deconvolution has a built in vector for separating haematoxylin (H) and diaminobenzidine (DAB) stainings. After colour deconvolution, H and DAB images are processed separately. By using five random test samples stained for Ki-67, suitable threshold levels for H and DAB were determined. These thresholds were used on both H and DAB images, respectively, and kept constant for the analysis of the main image dataset. Thresholding creates binary masks of H and DAB positive areas and the two areas may overlap. Binary masks were merged into a single result image. In the result image, the area of H-positive and DAB-negative pixels is pseudocoloured with green colour. The area of DAB-positive pixels regardless of H-status is pseudocoloured with red colour. The background, where both values are negative, is indicated with white colour.

The extent of staining is calculated as the total number of DAB-positive pixels divided by the union of the total number of H-positive pixels and the total number of DAB-positive pixels. The intensity of staining is calculated from DAB-positive area, as a mean pixel value of original DAB image. The mean intensity value is scaled to range from 0 to 100 percent.

### Statistical analysis

For statistical analyses, only the highest extent or intensity of Ki-67 staining was considered if multiple evaluable tissue cores were available for the same patient. Continuous Ki-67 extent and intensity values generated by the computer vision algorithm were grouped. There is no consensus for cut-off values in the literature, some studies use arbitrary values, some median and some divide data in tertiles [19,22]. In the current study we decided to split the patient series into three approximately equally sized groups according both to Ki-67 extent of staining tertiles and intensity tertiles. For evaluation of agreement between the visual and automated assessment of Ki-67 we also split the automated results into similar proportions of low, moderate and high expression as for the visual results. Frequency tables were analyzed using the chi-square test. The agreement between the visual and automated methods in the assessment of Ki-67 expression was estimated by percent-agreement and kappa-statistics. Since the categories of the variables are ordered as described before, a linearly weighted kappa value was used [28]. Life tables were calculated according to the Kaplan-Meier method. Distant disease-free survival was calculated from the date of the diagnosis to the date of detection of metastases outside of the locoregional area or to the date of death from breast cancer, if a patient died of breast cancer without distant metastases. Patients who died from an intercurrent disease were censored on the date of death. Survival curves were compared with the log-rank test. Multivariate survival analyses were done with the Cox proportional hazards model, entering the following covariates: automated assessment of Ki-67 extent of staining or visually assessed Ki-67 proliferation index, method of tumour detection, tumour size in centimetres, number of metastatic lymph nodes, histological grade, and age at diagnosis. The assumption of proportional hazards was ascertained by assessment of log minus log survival plots. All P values are two tailed.

## Results

### Automated Ki-67 expression assessment

**Figure 3:**
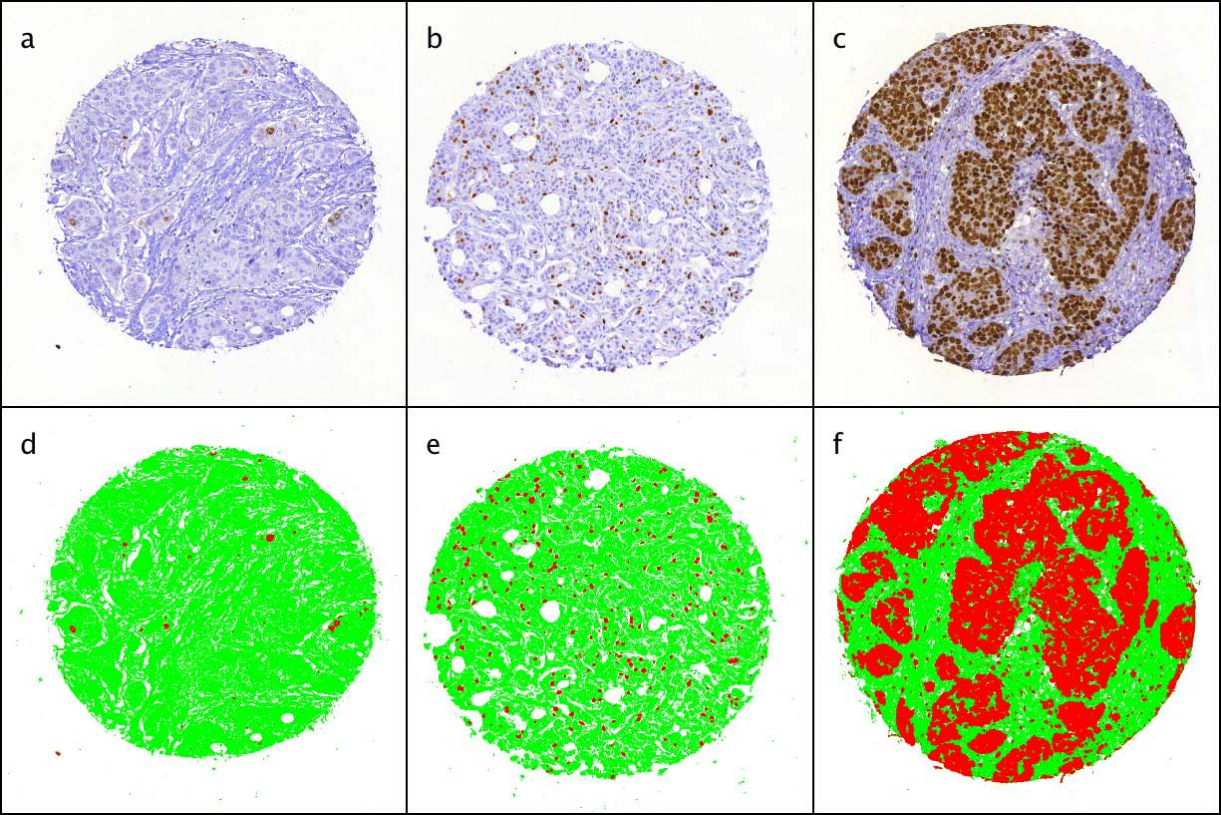
**Sample images of variable area of Ki-67 staining in FinProg series**. Low extent of staining (a), moderate extent of staining (b), high extent of staining (c), and corresponding result images from IhcJ macro (d, e, f).

A total number of 1648 TMA spot images from the 1334 subjects were analysed in 107 minutes. There were 251 subjects with duplicate, 21 with triplicate and 7 with quadruplicate cores among the readable cases. The average analysis time for single TMA spot was 3.9 seconds. The mean and median extent of staining for Ki-67 according to the computer vision analysis was 8.8% and 4.3% (range 0-95.3%) and the mean and median intensity was 52.5% and 53.2% (range 33.9-67.0%). Sample images are displayed in figure 3. For the purpose of statistical analysis, the patient series was split at tertiles into groups according to the automated assessment of Ki-67 extent of staining: 0 to 2.3 percent extent of staining was assigned to the low extent group, 2.4 to 6.3 percent extent of staining to the moderate extent group and 6.4 to 100 percent to the high extent group. For automated assessment of Ki-67 intensity the tertile thresholds were: low intensity group from 0 to 49.6 percent, moderate intensity group from 49.7 to 56.6 percent, and high intensity group from 56.7 to 100 percent.

### Visual assessment of Ki-67

According to visual assessment of Ki-67 staining, 7.7% of the patients were assigned to the negative expression group, 55.7% to the moderate expression and 36.7% to the high expression group.

### Association of automated assessment of Ki-67 expression with clinicopathological characteristics

When the patient series was split according to automated assessment of Ki-67 extent of staining into similar size groups as the visual Ki-67 results, 76% of the cases with high visually assessed extent had high automatically assessed extent, and none had low extent. Forty-six percent of the cases with negative visually assessed extent also had low automatically assessed extent, and only 2% had high extent (table 1). The percentage agreement was 87% and weighted kappa value 0.57 (table 1).

**Table 1:** Agreement between automatic and visual assessment of Ki-67 proliferation index (Kappa statistics, linear weights, Percentage agreement 87%, Kappa 0.57)

			Visual		
		Negative	Moderate	High	Total
	Low	46	53	0	99
Automatic	Moderate	51	554	114	719
	High	2	112	360	474
	Total	99	719	474	1292

For the main analysis, we examined the relationship between automated assessment of Ki-67 expression and clinicopathological variables by splitting the series according to automated assessment of Ki-67 tertiles. Patients younger than 35 years at the time of diagnosis had a significantly higher extent of Ki-67 staining than those diagnosed at a higher age (P = 0.0008) (table 2). Cancers detected within mammography screenings had lower automated assessment of Ki-67 extent than those detected outside of screening (P = 0.0001). Increasing primary tumour size is strongly associated with higher Ki-67 extent of staining (P < 0.0001), as is increasing histological grade (P < 0.0001). A higher number of metastatic axillary lymph nodes also associates with higher Ki-67 extent of staining (P = 0.01). There is a significant difference in distribution of automated assessment of Ki-67 extent among different histological types of breast cancer (P < 0.0001): in ductal carcinomas, higher extent is more frequent, whereas lower extent of Ki-67 staining predominates in lobular carcinomas. For molecular markers, high automated assessment of Ki-67 extent is strongly associated (all with P < 0.0001) with negative ER and PR expression, positive HER2 amplification and expression and high p53 expression. When molecular subtypes are considered, only Luminal A is associated with low automated assessment of Ki-67 extent, while other molecular subtypes tend to have higher Ki-67 extent of staining (P < 0.0001).

**Table 2:** Association of automated assessment of Ki-67 extent of staining with clinicopathological characteristics

Characteristic	Automated assessment of Ki-67 extent of staining			P
	Low N (%)	Moderate N (%)	High N (%)	
All tissue samples (N = 1334) *	406 (30)	446 (33)	482 (36)	
Ki-67 expression (visual)				
High	13 (3)	102 (22)	359 (76)	< 0.0001
Moderate	293 (41)	314 (44)	112 (16)	
Negative	82 (83)	15 (15)	2 (2)	
Not available	18 (43)	15 (36)	9 (21)	
Age at diagnosis (y)				
< 35	2 (6)	13 (36)	21 (58)	0.0008
35-50	101 (28)	120 (33)	141 (39)	
50-65	160 (36)	133 (30)	149 (34)	
>65	143 (29)	180 (36)	171 (35)	
Method of detection				
Mammography screening	102 (41)	81 (33)	66 (27)	0.0001
Other	298 (28)	355 (34)	403 (38)	
Not available	6 (21)	10 (34)	13 (45)	
Primary tumour diameter (cm)				
< 0.5	8 (44)	8 (44)	2 (11)	< 0.0001
0.5-1	82 (42)	66 (34)	49 (25)	
1-2	182 (34)	180 (34)	172 (32)	
2-5	104 (22)	156 (33)	212 (45)	
> 5	13 (24)	14 (25)	28 (51)	
Not available	17 (29)	22 (38)	19 (33)	
No. of positive axillary nodes				
0	276 (35)	253 (32)	264 (33)	0.0142
1-3	80 (27)	98 (33)	119 (40)	
4-9	26 (23)	45 (40)	41 (37)	
≥ 10	5 (16)	11 (35)	15 (48)	
Not available	19 (19)	39 (39)	43 (43	
Histological grade				
1	118 (52)	73 (32)	37 (16)	< 0.0001
2	122 (26)	184 (39)	171 (36)	
3	34 (12)	67 (23)	188 (65)	
Not available	132 (39)	122 (36)	86 (25)	
Histological type				
Ductal	275 (27)	342 (34)	402 (40)	< 0.0001
Lobular	87 (46)	67 (35)	36 (19)	
Special	44 (35)	37 (30)	44 (35)	
ER expression				
Positive	301 (35)	324 (38)	234 (27)	< 0.0001
Negative	69 (17)	102 (26)	228 (57)	
Not available	36 (47)	20 (26)	20 (26)	
PR expression				
Positive	254 (37)	270 (39)	171 (25)	< 0.0001
Negative	122 (21)	151 (27)	295 (52)	
Not available	30 (42)	25 (35)	16 (23)	
HER2 amplification				
Positive	34 (14)	75 (31)	134 (55)	< 0.0001
Negative	331 (33)	333 (34)	327 (33)	
Not available	41 (41)	38 (38)	21 (21)	
HER2 expression				
Positive	26 (13)	60 (29)	121 (58)	< 0.0001
Negative	333 (33)	340 (34)	324 (33)	
Not available	47 (36)	46 (35)	37 (28)	
p53 expression				
High	16 (7)	50 (22)	158 (71)	< 0.0001
Moderate	98 (27)	135 (37)	129 (36)	
Low	222 (39)	196 (34)	155 (27)	
Not available	70 (40)	65 (37)	40 (23)	
Molecular subtype				
Basal	11 (12)	15 (16)	69 (73)	< 0.0001
HER2+/HR-	14 (12)	33 (28)	70 (60)	
Luminal A	291 (38)	288 (37)	191 (25)	
Luminal B	19 (16)	37 (32)	60 (52)	
Five-marker negative	8 (19)	6 (14)	28 (67)	
Unclassified	9 (16)	18 (32)	29 (52)	
Not available	54 (39)	49 (36)	35 (25)	

* due to ties the number of samples in each tertile is not equal

### Uni- and multivariate analyses of distant disease-free survival

**Figure 4:**
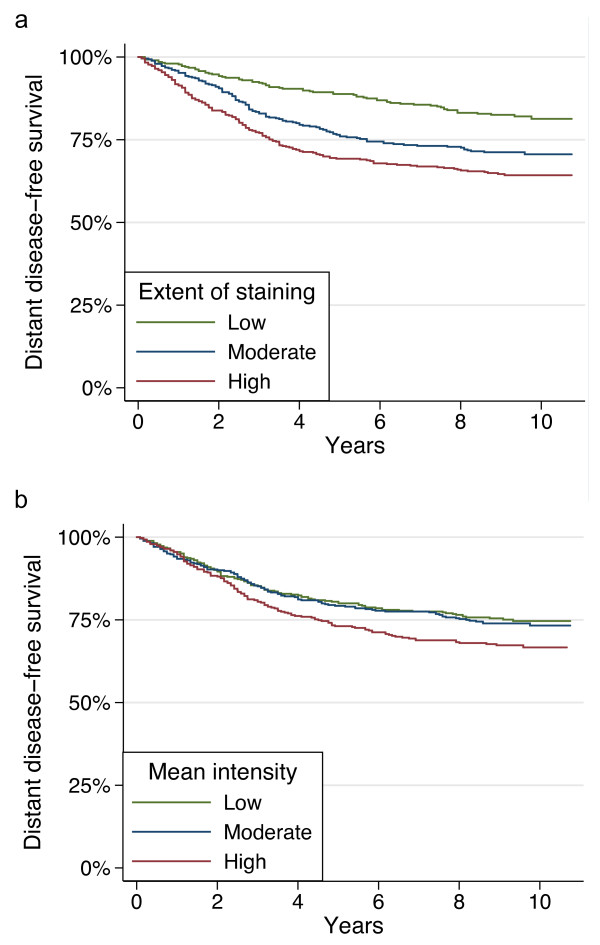
**Distant disease-free survival (Kaplan-Meier curves, whole dataset)**. by the grouped Ki-67 extent of stainings (a), and by the grouped Ki-67 staining intensities (b).

Increasing extent of Ki-67 staining as determined by image analysis is significantly associated with a decrease in distant disease-free survival (DDFS; figure 4a). Patients with a moderate automated assessment of Ki-67 extent of staining have a hazard ratio of 1.77 (95% CI 1.31-2.37) for distant recurrence, and those with the high automated assessment of extent of staining have a hazard ratio of 2.34 (95% CI 1.76-3.10), as compared to patients with a low automated assessment of Ki-67 extent of staining (table 3). The 5- and 10-year DDFS for the low Ki-67 extent group were 89% and 81%, respectively (table 4). DDFS for the moderate automated assessment of Ki-67 extent of staining group were 77% and 71%, and for the high automated assessment of Ki-67 extent group 69% and 64%.

**Table 3:** Cox uni- and multivariate distant disease-free survival analyses

Covariate (univariate analyses)	HR	95% CI	P
Ki-67 (Automated assessment of extent of staining)			
Low	1.00		
Moderate	1.77	1.31-2.37	< 0.0001
High	2.34	1.76-3.10	< 0.0001

Ki-67 (Visually assessed proliferation index)			
Negative	1.00		
Moderate	1.41	0.83-2.39	0.207
High	2.58	1.52-4.37	< 0.0001

Covariate (multivariate analyses)	HR	95% CI	P

Ki-67 (Automated assessment of extent of staining)			
Low	1.00		
Moderate	1.62	1.10-2.39	0.014
High	1.73	1.19-2.51	0.004
Tumour size*	1.23	1.14-1.34	< 0.0001
No. of positive axillary lymph nodes†	1.14	1.11-1.18	< 0.0001
Histological grade (grade 3 or 2 vs. 1)	2.17	1.36-3.47	0.001
Detection at mammography screening	0.50	0.32-0.78	0.002
Age at diagnosis			
< 50	1.00		
>50	1.18	0.90-1.55	0.221

Ki-67 (Visually assessed proliferation index)			
Negative	1.00		
Moderate	0.71	0.37-1.38	0.311
High	1.03	0.53-2.01	0.920
Tumour size*	1.25	1.15-1.35	< 0.0001
No. of positive axillary lymph nodes†	1.14	1.11-1.18	< 0.0001
Histological grade (grade 3 or 2 vs. 1)	2.36	1.46-3.80	< 0.0001
Detection at mammography screening	0.47	0.30-0.74	0.001
Age at diagnosis			
< 50	1.00		
>50	1.20	0.92-1.58	0.185

*Hazard provided per one centimetre of the longest diameter of the tumour

†Hazard provided per one metastatic node

**Table 4:** 5- and 10-year survival

Automated assessment of Ki-67 extent of staining	N	5-year survival (95% CI)	10-year survival (95% CI)
Low	334	89 % (85%-92%)	81% (77%-85%)
Moderate	303	77% (72%-80%)	71% (66%-75%)
High	305	69% (65%-73%)	64% (60%-69%)

In subgroup analysis according to histological grade, patients with grade 1 tumours and moderate automated assessment of Ki-67 extent had a hazard ratio of 1.05 (95% CI 0.35-3.22) and patients with high automated assessment of Ki-67 extent a hazard ratio of 4.63 (95% CI 1.83-11.75), as compared to patients with low automated assessment of Ki-67 extent. In patients with grade 2 tumours, the corresponding figures were 1.51 (95% CI 0.95-2.42) and 1.99 (95% CI 1.26-3.15). In the grade 3 subgroup, the results were 1.37 (95% CI 0.69-2.73) and 0.97 (95% CI 0.51-1.84). In the subgroup of patients with ductal breast cancer, the figures were 1.83 (95% CI 1.30-2.58) and 2.30 (95% CI 1.66-3.18), and in the lobular carcinoma subgroup, the corresponding hazard ratios were 1.40 (95% CI 0.67-2.93) and 2.29 (95% CI 1.06-4.95).

When extent of Ki-67 staining was determined visually, patients with a moderate visually assessed Ki-67 expression had a hazard ratio of 1.41 (95% CI 0.83-2.39) for distant recurrence, and those with a high visually assessed Ki-67 expression had a hazard ratio of 2.58 (95% CI 1.52-4.37), as compared to patients with a negative visually assessed Ki-67 expression.

For computer determined Ki-67 intensities, no statistically significant difference was observed between low and moderate intensity groups (figure 4b). The high intensity group was associated with significantly less favourable distant disease-free survival (HR = 1.34, 95% CI 1.04-1.73) compared to the low intensity group.

In a multivariate survival analysis, adjusted for tumour size, the number of positive lymph nodes, histological grade, method of detection and age at diagnosis, patients with a moderate automated assessment of Ki-67 extent of staining had a hazard ratio of 1.62 (95% CI 1.10-2.39) and those with high Ki-67 extent a hazard ratio of 1.73 (95% CI 1.19-2.51), compared to patients with low automated assessment of Ki-67 extent of staining (table 3). The visually determined Ki-67 expression was not retained as a significant prognostic factor if entered instead of the computer determined Ki-67 extent in the same multivariate model (table 3). Computer determined Ki-67 intensity was not significantly associated with DDFS in the multivariate model.

## Discussion

In the current report we describe the integration of an open source image analysis tool with a virtual microscopy platform. Computer determined extent of immunohistochemical staining of the extensively studied biomarker Ki-67 shows prognostic value comparable to visually assessed Ki-67 in a comprehensive series of patients with breast cancer.

The automated assessment of Ki-67 extent of staining was significantly associated with all the examined clinicopathological characteristics, including tumour size, number of positive lymph nodes, histological type and grade, oestrogen and progesterone receptor status, age at diagnosis, method of tumour detection, as well as molecular subtypes. These findings are in good agreement with previously reported results on the association between Ki-67 expression and clinicopathological factors [25,29]. The comparison of visual and automated assessment of Ki-67 expression showed only moderate agreement. The human observer may exclude non-tumour or stromal areas in the sample more effectively than the image analysis algorithm, which may explain part of the discrepancies. Also, the image analysis algorithm can include artefacts and staining errors. On the other hand, the human interpretation can vary due to the visual evaluation being done on several separate occasions. Visual thresholds may change between scoring sessions because of altered microscopy settings or reference spots with varying stain intensity. The observed variability between visual and automated methods mostly occurred between adjacent groups, only 2 patients with negative visual score were in the automatically assessed high extent group, and none of the patients with high visual score were classified by the automated analysis into the low extent group. The two totally discrepant cases were caused by partially folded TMA spot and falsely dyed spot. In general, automated method underestimated the extent of staining in samples with high stromal content or with just a few strongly positive tumor nuclei. The main causes for too high automated scores were out-of-focus samples, debris on the glass slide or positive staining of the tumor cytoplasm.

The analysis of distant disease-free survival shows that the automated assessment of Ki-67 extent of staining is a significant predictor of outcome in breast cancer. When compared to low extent of Ki-67 staining, moderate and high extent of staining is associated with hazard ratios of 1.77 and 2.34 for distant recurrence during the follow-up period. These results are in line with previous meta-analyses, where the pooled hazard ratios for disease-free survival (DFS) associated visually determined Ki-67 overexpression have been 1.93-2.18 [19,22].

When other variables are taken into account in the multivariate survival analysis, the automated assessment of Ki-67 extent of staining remains as a significant prognostic factor with hazard ratios of 1.62 and 1.73 for moderate and high extent of staining groups, respectively. Thus, the automated assessment of Ki-67 extent of staining is an independent predictor of patient outcome after adjustment for established clinicopathological factors. Also this is in agreement with results of meta-analyses, where a pooled DFS hazard ratio for Ki-67 overexpression in multivariate analysis was reported to be 1.76-1.84 [19,22]. In the current study, the visually determined Ki-67 failed to reach significance in the multivariate model, possibly due to previously discussed variability in human observer assessment. The automated Ki-67 intensity assessment was of limited prognostic value. This could partly be explained by the difficulties in quantification of the diaminobenzidine staining, due to a previously described non-linear relationship between the amount of antigen and the staining intensity [30].

The strengths of this study include that we analysed a large unselected breast cancer series with long follow-up period. The software and algorithms that were utilized are open source and integrated into a virtual microscopy platform. They could be made freely available as a software service on a public web site. Examples of this approach have recently been published and represent a promising methodology for standardization of quantitative immunostaining assessment and fluorescence in situ hybridization (FISH) signal counting [17,26]. This method could also be useful tool in routine breast cancer diagnostic pathology of prognostic and predictive factors. Even though the usage of TMA slides in this domain has shown promising results [31], these factors are mainly assessed from whole slide sections. Our approach can, however, be applied also to whole slide sections via either taking digital snapshots of regions of interest, or in case of virtual whole slides, the area to be analyzed can be selected manually.

A weakness of the algorithm proposed in our study is the need for manual adjustment of threshold levels before starting the batch analysis. A constant threshold level for the image analysis algorithm seems acceptable if similar tissue processing and staining protocols are applied throughout the whole specimen series, as in the current study. Another weakness of the current computer vision approach is that also stromal components of tissue samples were included in the automated assessment. This affects the distribution of the extent of staining as compared to studies that have excluded tumour stroma and calculated the proportion of stained cells in the tumour parenchyma only. An approach that does not exclude stroma might be acceptable for analysis of tissue microarrays that have been constructed to mainly contain tumour tissue. However, also in the TMAs the ratio of stroma to tumour epithelium can vary according to tumour grade and histological type, which can affect the extent of staining. Therefore we performed subgroup analysis according to histological grade and type, as well as adjusted for these possible confounders in a multivariate survival model. The automated assessment of Ki-67 extent of staining was a significant prognostic factor in all subgroups, except for in the group of patients with poorly differentiated tumours. This is in line with previous results showing a lack of prognostic value of Ki-67 in grade 3 tumours [32]. For analysis of individual samples and whole slide surgical tumour samples within a routine diagnostic setting, an image analysis method for excluding stroma would be needed. Examples of such method have been described in commercial systems [15,16]. Also, a method that segments and analyses tumour nuclei only might be better suited for the Ki-67 antigen, which mainly is expressed in the cell nuclei. However, algorithms that segment tumour nuclei require an optimal nuclear counterstain, which can be hard to achieve in practice. On the other hand, a recent study showed that also cytoplasmic and membranous expression of Ki-67 is of prognostic value in breast cancer [33].

## Conclusions

We conclude that the extent of Ki-67 staining determined by automated image analysis algorithm is an independent predictor of survival in breast cancer based on multivariate survival analysis. In univariate analysis, the automated assessment of Ki-67 extent of staining yields comparable results to the visual Ki-67 assessment. The current approach could be utilized in screening of prognostic biomarkers in large series of tissue microarrays, by integration with whole slide microscopy imaging and virtual microscopy.

## Declaration of competing interests

The authors declare that they have no competing interests.

## Authors' contributions

JK participated in the design of the study, assembled the image analysis system, performed data analysis and interpretation, statistical analysis and manuscript preparation. ML wrote the code for the virtual microscopy platform, participated in data acquisition and analysis, and manuscript revision. HJ and JL devised the nationwide FinProg breast cancer study. HJ supervised the FinProg study, and participated in manuscript revision. KH, TT-H, VK and LS participated in data acquisition and manuscript revision. TL assembled the tissue microarrays and participated in data acquisition and manuscript revision. HS performed staining of the samples and visual interpretation of the staining results, and participated in manuscript revision. JI supervised the laboratory work, contributed to the design of the image analysis system, and participated in data acquisition and manuscript revision. JL designed the study, performed data analysis and interpretation, statistical analysis and manuscript preparation. All authors read and approved the final manuscript.

## Role of the Funding Source

The study sponsors had no involvements in the study design, in the collection, analysis and interpretation of data; in the writing of the manuscript; and in the decision to submit the manuscript for publication.

## Pre-publication history

The pre-publication history for this paper can be accessed here:

http://www.biomedcentral.com/1472-6890/11/3/prepub
